# Pancreatic cancer risk prediction using deep sequential modeling of longitudinal diagnostic and medication records

**DOI:** 10.1016/j.xcrm.2025.102359

**Published:** 2025-09-16

**Authors:** Chunlei Zheng, Asif Khan, Daniel Ritter, Debora S. Marks, Nhan V. Do, Nathanael R. Fillmore, Chris Sander

**Affiliations:** 1 VA Boston Healthcare System, Boston, MA, USA; 2 Harvard Medical School, Boston, MA, USA; 3 Department of Computer Science, Cornell University, Ithaca, NY, USA; 4 Boston University School of Medicine, Boston, MA, USA; 5 Broad Institute of MIT and Harvard, Boston, MA, USA; 6 Ludwig Center at Harvard, Boston, MA, USA

**Keywords:** pancreatic cancer, risk stratification, deep learning for healthcare, machine learning for healthcare, AI for medicine, early detection of cancer

## Abstract

Pancreatic ductal adenocarcinoma (PDAC) is a rare, aggressive cancer often diagnosed late with low survival rates, due to the lack of population-wide screening programs and the high cost of early detection methods. To enable early detection of high-risk individuals, we develop a transformer-based model trained on longitudinal Veterans Affairs electronic health record (EHR) with 19,426 PDAC cases and ∼15.9 million controls. Our model combines diagnostic and medication trajectories to predict PDAC risk within a 6-, 12-, and 36-month assessment window. Incorporating medication significantly improved performance; among the top 1,000–5,000 highest-risk patients in a cohort of 1 million patients, 3-year PDAC incidence is 115–70 times higher than a reference estimate based on age and sex alone. Furthermore, analysis of most predictive features highlights the role of events such as chronic inflammatory conditions and specific medications on overall PDAC risk. Our work provides an AI-driven identification of high-risk individuals, with a potential to improve early detection, enhance patient care, and reduce healthcare costs.

## Highlights

• PDAC survival is low when diagnosed late, making risk prediction tools very useful
• AI trained on large-scale EHR diagnosis and medication data improves PDAC risk prediction
• Results show significant SIR improvements in high-risk cohorts
• Model interpretation highlights clinical risk factors guiding predictions

## Graphical abstract

**Figure:**
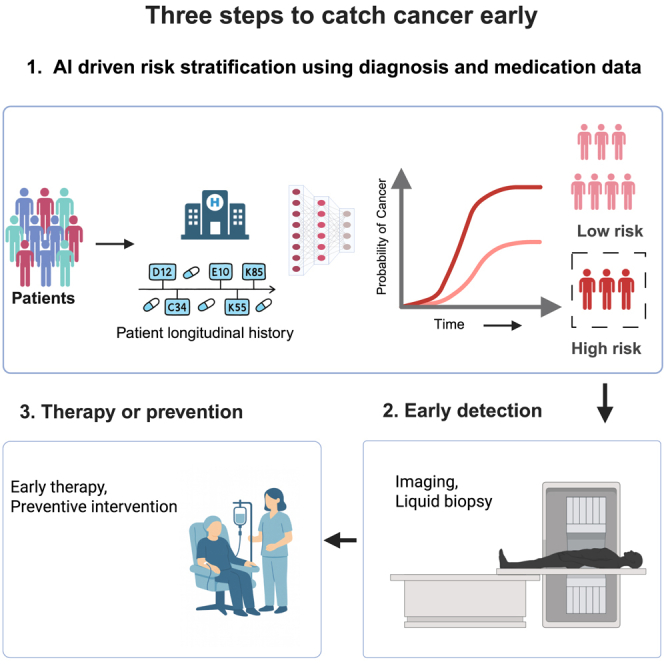

## Teaser

Zheng et al. propose a transformer-based AI model trained on longitudinal EHR to predict pancreatic cancer risk within 6, 12, 24, and 36 months. The model achieves superior performance over age- and sex-based incidence baselines and is available as open-source software designed for real-time clinical integration to enable earlier detection.

## Introduction

Pancreatic ductal adenocarcinoma (PDAC) is one of the most challenging cancers to diagnose with a poor prognosis, often detected at advanced stages when curative treatment options are limited. Its increasing incidence makes it one of the leading causes of cancer-related deaths worldwide.^1^^,^^2^ Approximately 80% of patients with pancreatic cancer are diagnosed with locally advanced or distant metastatic disease and have poor survival rates (only ∼12% survive for 5 years).^3^ In contrast, for the ∼20% of patients with early-stage disease (stage IA), survival rates are much higher (∼80% of these survive for 5 years, according to the US Veterans Affairs (US-VA) cancer registry^4^). These patients can be effectively treated by a combination of surgery, chemotherapy, and radiotherapy.^5^ Therefore, identifying risk factors and improved detection at early stages could significantly improve outcomes and reduce mortality from this aggressive malignancy.

### Early risk models on selected cohorts

Early detection programs can improve patient outcomes, but universal implementation is currently impractical due to the risk of false positives and high cost of screening for some cancer types, such as PDAC. An affordable selective screening approach targets high-risk individuals with an acceptable low rate of false positives. Previous works on selected (but relatively small) cohorts have developed risk models using factors such as family history, lifestyle, environmental, and genetic variants^6^^,^^7^^,^^8^^,^^9^^,^^10^^,^^11^ and identified individuals at increased risk of PDAC when such data were available.^12^

### Using real-world EHR data in predictive AI models

Electronic health records (EHRs) offer a rich and generally available source of patient data, including diagnoses, medications, and laboratory results, that can support population-wide risk prediction. Prior works have demonstrated the application of machine learning (ML) in EHR systems, such as BEHRT, a transformer model specifically designed for general prediction of future diagnoses of patients.^13^ Similar ML approaches have been explored in other recent works.^14^^,^^15^^,^^16^^,^^17^ Training specific ML prediction models on routinely collected large-scale real-world EHR data could enable targeted screening and preventive interventions for patients with high-risk PDAC. Recent works have used EHR data to develop several PDAC risk prediction models,^3^^,^^18^^,^^19^^,^^20^^,^^21^^,^^22^ which can be used to identify high-risk cohorts.

### Medication data for improved risk prediction

Most cancer risk prediction models based on EHR data predominantly rely on diagnosis codes alone, and only a few use other forms of data types, such as medication records.^3^^,^^18^^,^^19^ Diagnosis codes, especially in the US healthcare system, are influenced by billing incentives and reimbursement practices, which can lead to “billing pollution,” where differential or secondary diagnosis codes are upcoded to justify costly procedures or maximize reimbursement.^20^^,^^21^ This data noise can result in a somewhat inaccurate representation of a patient’s state of health. In contrast, medication data plausibly provide more objective information as prescriptions are based on well-trained physicians’ decisions and are more likely to represent objective clinical necessity rather than financial motivation. In addition, use of medications can provide direct information on disease risk due to known preventive effects or negative side effects.

### Drug effects on cancer risk

The usage of certain medications has been linked to altered cancer risk, with either positive or negative effects, e.g., consider omeprazole, a proton pump inhibitor commonly used to treat gastroesophageal reflux disease. Recent epidemiological studies have raised concerns based on a potential association between long-term use of omeprazole and an increased risk of developing PDAC.^22^^,^^23^^,^^24^ This association is attributed to hypergastrinemia, a condition characterized by elevated levels of the hormone gastrin, which has been shown to stimulate the growth of pancreatic cancer cells via interaction with the cholecystokinin B receptor. An opposite example is metformin, commonly used to treat type 2 diabetes, which has been associated with a decreased risk of PDAC.^25^ Metformin works by lowering blood glucose levels^26^^,^^27^ and also reduces levels of inflammatory markers,^28^^,^^29^ both of which are factors that can contribute to cancer development. While such associations with altered risk may be influenced by various confounding factors, medications may provide non-redundant information about cancer risk.

### Non-sequential versus sequential risk models

Previous risk prediction efforts have primarily relied on a pre-identified set of diagnosis codes as input features, overlooking valuable longitudinal information within EHR and the sequential nature of clinical events. Recently, a PDAC risk model (“PRism”)^3^ trained on a large-scale TriNetX^30^ database incorporated composite features, including diagnosis records, medication history, and laboratory results, to encode each patient’s history as a fixed 87-dimensional vector, which was then used to train multilayer perceptron and logistic regression models. The choice of input features in PRism does not model the sequential ordering and timing of events explicitly. Unlike PRism, our AI tool uses deep sequential learning, explicitly capturing the sequential order and timing of clinical events for more nuanced PDAC risk prediction.

### Combination model trained on medication and diagnostic data

**Figure 1:**
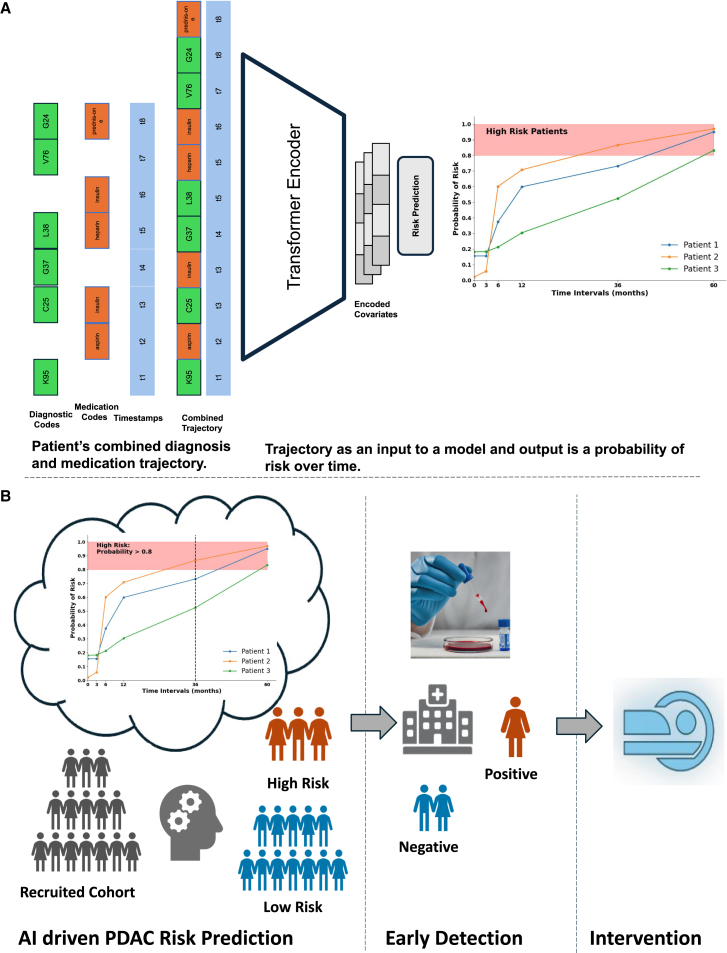
AI tool for risk prediction (A) Example of input trajectory based on the combination of diagnostic (ICD) and medication (RxNorm) codes. A trajectory represents the patient’s medical history, encompassing diagnoses and prescribed medications along with respective timestamps. The combined trajectory is given as input to the AI prediction tool, which uses a transformer encoder to extract latent risk factors. These factors are then fed to a risk prediction model that calculates the probability of cancer risk over different time intervals. (B) Surveillance program that uses the AI tool to identify high-risk patients. Identified high-risk cohorts can benefit from early detection technologies and possible healthcare interventions.

Previously, CancerRiskNet^31^ introduced a transformer-based sequential model for PDAC risk prediction, using only diagnosis codes. Here, we extend this architecture to longitudinal sequences of both diagnosis and medication codes, allowing the model to learn a shared encoded space that captures both clinical diagnostic histories and prescribed drugs over time. Our model utilizes self-attention to capture interactions among disease and medication events, while accounting for temporal spacing using positional encoding based on relative event timings. Developed and tested on a large-scale US-VA dataset, this model is an approach to integrate diagnostic and medication data for PDAC risk prediction. Our work facilitates the identification of high-risk cohorts who could benefit from a three-step clinical program—prediction, detection, and intervention (Figure 1B)—to improve patient outcomes.

## Results

### PDAC risk prediction using diagnosis and medication data

#### Datasets for model development and evaluation

**Figure 2:**
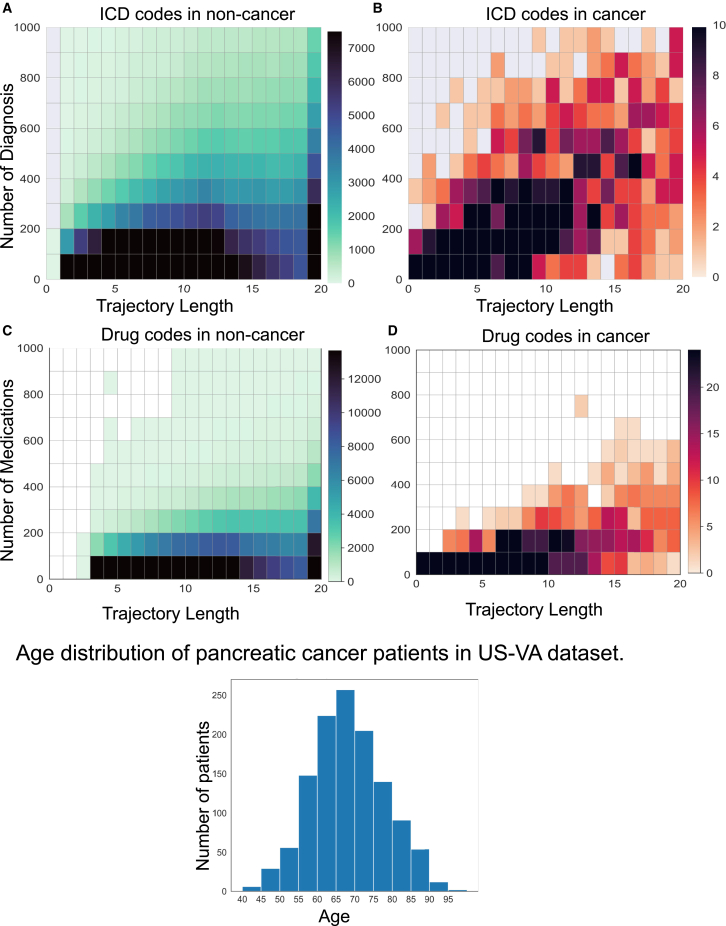
The density of medications and diagnosis codes for different data trajectory lengths Cancer patients (A and C) and non-cancer patients (B and D). The intensity of color is a number of patients in a bin. For non-cancer patients, patients with less than 2 years of follow-up were removed.

We use the US-VA database to construct training and evaluation datasets, based on a total of 15,926,415 patients, including 19,426 cases of pancreatic cancer. Overall data statistics including the data splits used for model development and evaluation are in Table 1 and Figure 2. We use each patient’s time-based trajectory of International Classification of Diseases (ICD) codes for diagnoses and active ingredients from prescription medications. Consistent with the approach in earlier work,^31^ we restrict ICD codes to three-character categories within the ICD hierarchy to focus on fairly broad diagnostic groups. Timestamps associated with the diagnosis or medication event codes are incorporated in the model as positional encodings to account for the temporal aspect of patients’ clinical histories.

**Table 1:** Patient records in the US-VA dataset for model training and evaluation

Development dataset	Overall	Non-cancer	Cancer
***n* (number of patients)**	210,848	192,722	18,126

**By gender, *n* (%)**			

Female	28,071 (13.3)	27,566 (14.3)	505 (2.8)
Male	182,774 (86.7)	165,153 (85.7)	17,621 (97.2)

**By race, *n* (%)**			

Asia/Pacific Islander	3,066 (2.1)	2,917 (2.2)	149 (1.0)
Black	25,708 (17.3)	22,670 (16.9)	3,038 (20.6)
Native	1,159 (0.8)	1,062 (0.8)	97 (0.7)
White	119,061 (79.9)	107,582 (80.1)	11,479 (77.8)

**ICD diagnosis trajectories**			

Lengths (# events), median [Q1, Q3]	91.0 [15.0, 298.0]	82.0 [13.0, 283.0]	195.0 [73.0, 433.0]
Time (years), median [Q1, Q3]	7.0 [2.0, 14.0]	8.0 [2.0, 14.0]	7.0 [3.0, 12.0]

**Medication trajectories**			

Number of prescriptions, median (# events) [Q1, Q3]	50.0 [14.0, 129.0]	48.0 [13.0, 126.0]	73.0 [29.0, 152.0]
Time (years), median [Q1, Q3]	8.0 [3.0, 14.0]	8.0 [3.0, 15.0]	7.0 [2.0, 12.0]

**Evaluation dataset**			

***n* (number of patients)**	987,693	986,393	1,300

**By gender, *n* (%)**			

Female	141,224 (14.3)	141,179 (14.3)	45 (3.5)
Male	846,468 (85.7)	845,213 (85.7)	1,255 (96.5)

**By race, *n* (%)**			

Asia/Pacific Islander	15,544 (2.3)	15,534 (2.3)	10 (0.9)
Black	114,733 (16.6)	114,512 (16.6)	221 (20.6)
Native	5,449 (0.8)	5,445 (0.8)	4 (0.4)
White	553,383 (80.3)	552,544 (80.3)	839 (78.1)

**Trajectories-ICD diagnoses**			

Lengths (# events), median [Q1, Q3]	83.0 [13.0, 282.0]	82.0 [13.0, 282.0]	207.0 [78.0, 458.5]
Time (years), median [Q1, Q3]	8.0 [2.0, 14.0]	8.0 [2.0, 14.0]	7.0 [3.0, 12.0]

**Trajectories-medications**			

Number of prescriptions, median (# events) [Q1, Q3]	48.0 [13.0, 126.0]	48.0 [13.0, 126.0]	78.0 [31.0, 167.0]
Time (years), median [Q1, Q3]	8.0 [3.0, 15.0]	8.0 [3.0, 15.0]	7.0 [2.0, 12.0]

US-VA data include patient visits in both inpatient and outpatient settings across the nation. The dataset for model development has 18K cancer cases and a randomly selected set of 193K controls. The dataset for model evaluation (also called held-out dataset in STAR Methods) has a subset of 1,300 cancer cases and, to reflect the balance in real-world data, 986K controls. Q1, first quantile and Q3, third quantile.

To train the AI model, we subsample trajectories of fixed lengths for each patient, starting at the first recorded event and ending at various endpoints. This subsampling approach ensures a more comprehensive modeling of patient risk, as it captures periods when specific symptoms develop. As a result, the same patient can have trajectories representing variations in cancer risk over time. These variations can occur for a number of reasons such as changes in lifestyle, disease states, or the use of specific medications. This approach allows us to interpret each trajectory as a realistic and dynamic representation of a patient’s health over time.

#### Risk prediction model

The architecture of the risk prediction model is based on the framework described earlier.^31^ The earlier implementation only uses diagnostic codes. The current model takes as input combined trajectories of medication and diagnostic codes, along with their respective timestamps, ending at the time of prediction. Initially, all events in a given trajectory of clinical data items are encoded into a fixed-length representation using an embedding layer. The timestamp information is incorporated in the form of relative positional encoding of events, which, when combined with the embedding, provides a time-dependent encoding of trajectories. These encodings are then input into a transformer model, which uses a self-attention mechanism to learn interdependencies between all codes within a trajectory, both diagnostic and medication. The transformer’s output is aggregated along the time dimension to produce a fixed-length latent representation of a given input trajectory. This representation is subsequently fed into a supervised risk regression model, which predicts the probability of cancer occurrence within five time intervals: 0–3, 0–6, 0–12, 0–36, and 0–60 months. Further details are in the STAR Methods.

#### Evaluation of prediction performance

Using different data types, including patient ICD codes, medication usage, and their combination, we trained three models and then evaluated their performance using four metrics: AUROC, positive predictive value, incidence ratio, and standardized incidence ratio (SIR). Further details on each metric are in the STAR Methods. The SIR is particularly relevant for developing a realistic surveillance program, as it compares the model’s predictions to standardized incidence rates from population-wide studies conditioned on age and gender. A SIR of 1 indicates that a model prediction is equivalent to frequency estimates based on population-wide studies, while higher values indicate better predictive performance. Considering the high cost of surveillance programs, these are currently most realistic for a relatively small number of patients at a very high risk of pancreatic cancer. We, therefore, select the top 1,000 high-risk patients from a cohort of 1 million and report the SIR scores for different models.

The primary results are based on a held-out retrospective cohort for a model trained with a three-month exclusion window; performance on the 10% development-set test split is in Figure S8. We also evaluated models trained with no exclusion (Figure S4) and with a 12-month exclusion window (Figure S5). For the SIR, baseline incidence rates are derived from the US-VA cohort, and comparison with rates derived from the Surveillance, Epidemiology, and End Results (SEER) database, which offers estimates of nationwide incidence rates, are in Figure S7. Finally, the calibration curve for the 36-month window model is in Figure S6.

#### Model trained on combination data with data exclusion achieves improved performance

**Figure 3:**
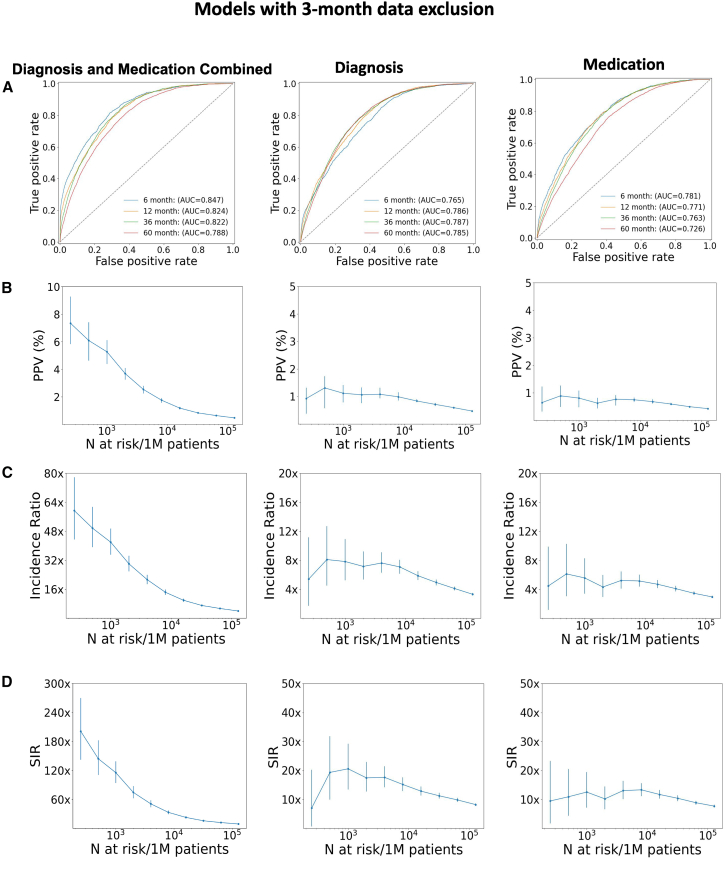
Comparison of the performance of model trained with a 3-month data exclusion window for the 36-month prediction interval: diagnostic data only, medication data only, and combination of both Left: model trained on the combination of diagnosis and medication data. Middle: trained on diagnosis data only. Right: model trained on medication data only. (A) Area under receiver operating characteristics (AUROC) performance curve for different prediction time intervals. (B–D) (B) Positive predictive value (PPV), (C) incidence ratio, and (D) standardized incidence ratio (SIR) for the *N* highest-risk patients out of 1 million patients. Data in plots represented as mean ± confidence interval. The curve can be used to choose a decision threshold for realistic implementations.

The ICD codes appearing shortly before the diagnosis of cancer can represent quasi-symptoms that would be easily interpreted by a clinician. To determine whether the model is primarily based on these quasi-symptoms or learns non-trivial covariates predictive of cancer, we trained models with and without a 3-month data exclusion window. The data exclusion involves removing all ICD codes and medications assigned within 3 months prior to the diagnosis of pancreatic cancer. With this data exclusion, compared to models from either ICD or medication alone, the combination model performed best (for each of the “cancer within k months” intervals). For instance, for the combined model, the area under the receiver operating characteristics (AUROC) is 0.822 (95% confidence interval [CI]: 0.812–0.835) for the 36-month prediction interval, while the diagnosis and medication models have lower AUROCs of 0.789 (95% CI: 0.782–0.793) and 0.763 (95% CI: 0.758–0.765), respectively (Figure 3A). As expected, the scores of models trained with data exclusion are lower when compared to the model without exclusion (see Figure 3S), which can be attributed to the absence of quasi-symptoms. The technical details of evaluation metrics and CI are included in STAR Methods.

**Figure 4:**
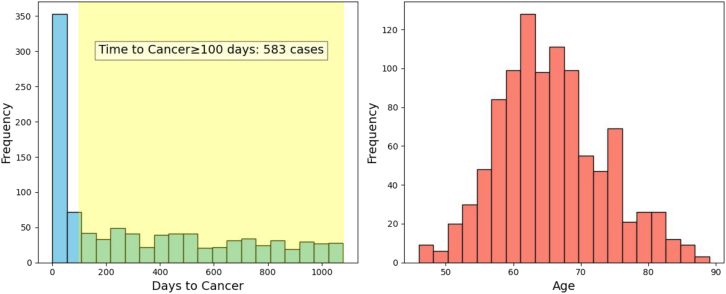
Characteristics of highest-risk subpopulation using a model trained with a 3-month data exclusion window for the 36-month prediction interval (*N*=1,000) (Left) Patients who were predicted to be at high risk and eventually got cancer are sampled to determine the time difference between the end of the disease trajectory (time point of prediction) and the actual cancer diagnosis (*x* axis). *y* axis: frequency of patients in the respective time gap. The frequency at larger time differences (yellow) indicates to what extent the model can correctly predict cancer diagnosis at larger time intervals before the actual diagnosis. (Right) Age distribution in the sampled high-risk cohort indicates good coverage.

With our focus on the highest-risk patients to be nominated for moderate-size affordable surveillance programs, we evaluate model performance (computed on the withheld test set) as a function of *N* highest-risk patients out of 1 million real-world all-comers. The standardized incidence ratio (SIR), which quantifies model performance relative to a simple model based only on incidence population statistics for a given gender and age bracket, decreases as one goes down the list of *N* patients ranked by predicted risk (Figure 3C). Baseline incidence rates for the SIR were derived from the Veterans Affairs (VA) Corporate Data Warehouse; we also analyze results using population-based incidence rates from SEER (Figure 4S; Table 1S). The curve reflects the tendency of generally higher accuracy at smaller *N* (except at very small *N* where small sample size results in higher uncertainty intervals). It can be used to make a particular choice of decision threshold such that *N* represents a conservative choice that minimizes false positives and optimizes performance by restricting the number *N* of high-risk patients nominated for surveillance. In a real-world application, *N*-threshold should be selected where the CI is low and stable, ensuring a balance between the cost and practical requirements of a clinical surveillance program targeting the highest-risk individuals.

To address the question of when in a prediction interval cancer is likely to occur, we evaluate, as an example, the actual time to cancer (in the test set) for *N* = 1,000 highest-risk patients (Figure 4). In this example, 37% of the highest-risk patients have cancer occurrence after more than 100 days. This kind of evaluation can be a guide to choice of tests and follow-up visit schedules in the design of surveillance programs.

#### Interpretation of the most predictive features provides plausible clinical hypothesis

To highlight the most relevant features driving the prediction of the risk model (attribution analysis), we applied the Integrated Gradients (IG)^32^ method to a set of high-risk patient trajectories. Features consisting of ICD codes and medications were ranked by their IG scores, with the top 10% identified as most predictive (details in STAR Methods; list in Table 2).

**Table 2:** Top ten most predictive diagnosis and medication codes using a model trained with a three-month data exclusion window for the 36-month prediction interval

Rank	Diagnosis codes	Medication codes
1	essential (primary) hypertension	lisinopril
2	non-insulin-dependent diabetes mellitus, insulin-dependent diabetes mellitus, other specified diabetes mellitus	simvastatin
3	disorders of lipoprotein metabolism and other lipidemias, other metabolic disorders, disorders of glycoprotein metabolism	hydrochlorothiazide
4	acute pancreatitis	aspirin
5	chronic ischemic heart disease	metformin
6	psoriasis, parapsoriasis, pityriasis rosea other papulosquamous disorders, other dermatitis	hydrocortisone
7	benign neoplasm of other and ill-defined parts of digestive system	nitroglycerin
8	other chronic obstructive pulmonary disease	docusate
9	polyarthrosis, other arthrosis, coxarthrosis	insulin
10	glaucoma, other congenital malformations of eye	vitamin B12

We conducted an attribution analysis on high-risk patients and listed the most predictive diagnosis (left) and medication (right) codes for high-risk patients as identified by our model.

Essential hypertension and diabetes have widely been associated with cancer risk,^33^ while lipoprotein metabolism disorders link to disrupted lipid metabolism.^34^ Conditions such as acute pancreatitis^35^ and chronic inflammatory conditions such as ischemic heart disease as well as chronic obstructive pulmonary disease are linked to sustained inflammation.^36^ These diagnoses include several metabolic, cardiovascular, and inflammatory conditions, which are known to be associated with high risk of pancreatic cancer.

Metformin, prescribed medication for type 2 diabetes, and insulin, used for blood glucose regulation, are both associated with elevated glucose levels known to increase risk of cancer.^37^^,^^38^ An association between the initiation of antidiabetic and anticoagulant medications and the diagnosis of pancreatic cancer in a 2-year pre-diagnosis time window was also reported in a study of controlled cohorts of nurses and health professionals.^39^ Aspirin and simvastatin, prescribed in cardiovascular or chronic inflammation conditions, may influence cancer risk through their anti-inflammatory effects.^40^^,^^41^ These medications likely indicate underlying metabolic or inflammatory conditions that may increase the likelihood of future cancer development, suggesting their role as markers of disease progression rather than direct causative agents.

These interpretations are to be considered with caution, as the IG method does not provide isolated effects of individual features; instead, it measures the influence of each feature change within the context of other features in the input vector. This implies that the predictive power of features emerges from their interdependencies rather than their independent contributions.

#### Evaluation in subpopulations reveals demographic differences in performance

AI models can be biased toward specific subpopulations based on the distribution of samples from different groups within the training dataset.^42^ For instance, if the training data predominantly include samples from one demographic group, the model is likely to perform better when evaluated on that specific group. Investigating such biases is important for assessing whether a model performs comparably across all subgroups.

**Figure 5:**
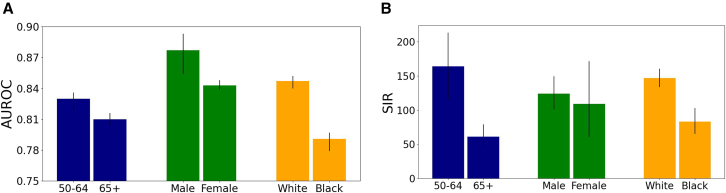
Evaluation of prediction performance in subpopulations using a model trained with a 3-month data exclusion window for the 36-month prediction interval (A) AUROC and (B) SIR, stratified by race, gender, and age. Data in plots represented as mean ± confidence interval. Notably, the SIR score is higher for the White subpopulation, as well as for male patients, and individuals in the 50–64 age group. The observed differences in performance across subpopulation groups can be attributed to biases in the dataset, as it consists of a higher proportion of White patients compared to Black patients, more male patients compared to female patients, and a greater number of individuals in the 50–64 age group compared to the 65+ age group, as detailed in Table 1*.*

We, therefore, stratified the evaluation dataset and validated the model on subpopulations defined by race and sex. The AUROC for White patients in a 12-month prediction window is 0.901 (95% CI: 0.897–0.904), compared to 0.870 (95% CI: 0.860–0.880) for Black patients. For male patients, it is 0.892 (95% CI: 0.888–0.896), compared to 0.900 (95% CI: 0.883–0.913) for female patients. Moreover, when examining the 1,000 high-risk cohorts, we observed that the SIR for White patients is higher than that for Black patients, and the SIR for male patients is higher than that for female patients. There are more White patients and more male patients in the database (Table 1), contributing to the observed performance disparities for the models trained on all patients. These gaps highlight the model’s bias plausibly resulting from the uneven representation of subpopulations in the dataset (Figure 5).

## Discussion

### Combination model trained on diagnostic and medication trajectories

This work highlights the information value of combining diagnosis and medication data in patients’ health histories using a transformer neural network for risk prediction of pancreatic cancer. Medication data carry information complementary to diagnosis codes and help address the hard-to-detect and hard-to-correct upcoding of diagnosis codes, as medication codes are less likely to be influenced by finance-motivated billing practices.^20^ In future, application of natural language processing of clinical notes to filter diagnosis codes and ML-learned feature selection may be useful to mitigate billing-related noise in EHR datasets. In addition, additional patient data such as blood test results and, in future, widely available genetic information will probably further enrich the model’s ability to learn from the most informative combinations of risk factors in a real-world population.

### Sequential modeling of EHR trajectories

By using a sequential model, the AI tool makes use of temporal dependencies between health events. Our results indicate that a sequential model, as opposed to a “bag-of-events” model, can capture temporal contributions of and informational synergy between disease and medication events, leading to improved performance in predicting cancer risk compared to non-sequential models (Figure S3).

### Focus on large real-world patient data and the highest-risk bracket

There is an informational trade-off between large real-world EHR datasets and specialized data from small specifically recruited cohorts. This study focuses on large real-world patient datasets in contrast to pioneering earlier prediction models in smaller controlled cohort studies, which have developed risk prediction models using carefully acquired well-defined risk factors such as family history, genetic markers, unintended weight loss, and systematic changes in fasting glucose levels.^6^^,^^7^^,^^8^^,^^9^^,^^10^^,^^11^ However, such valuable information is not generally available in the EHRs of all-comers in large populations. To cast a wider net, this study used EHRs from real-world populations based only on generally available information in health records, such as diagnoses and prescriptions.

In a trade-off that balances cost and benefit of surveillance programs, we focus on the selection of high-risk cohorts, which is only a small fraction of the large total population: patients at very high risk for the cancer with the highest probability of accurate prediction (lowest false positive prediction rate), for whom expensive screening tests are justified and affordable. For example, from all patients in a hospital system aged 50 years or older, out of 1 million patients on whose EHRs the prediction tool is applied, we propose to identify a subpopulation of a few thousand highest-risk patients and focus on a 12-month prediction interval. E.g., for 1,000 patients at highest risk, the result of SIR = 115 for a model trained with a 3-month exclusion window (not using data from a 3-month interval before cancer occurrence) quantifies how much better the AI tool is in identifying high-risk patients than usage of a background model that uses population-wide incidence rates, only using age and gender information. The tool thus may have concrete utility as a decision-support tool, aiding clinicians in an early detection program in a large hospital system.

### Prediction performance in subpopulations

We have also evaluated the prediction performance of the inclusively trained model in different race groups and highlight potential biases to be addressed in future work. Our retrospective (on a withheld test set) evaluation of the PDAC risk prediction model across different race groups highlights the potential impact of inherent biases in data distribution. Specifically, the model trained on the entire population had a lower AUROC score of 0.82 for the Black ethnic group compared to 0.85 for the White ethnic group, a lower score of 0.86 for male subpopulation compared to 0.88 for female subpopulation, and a higher score of 0.86 for patients aged 50–64 compared to 0.82 for patients aged 65+ (Figure 4). These disparities in performance can plausibly be attributed to inherent differences in the training data distribution, which leads to biases in prediction performance. For instance, there are more White than Black patients in the EHR database (Table 2), so disease and medication data items corresponding to White are more likely to be reflected in the parameters of the trained model. In addition, differences in the type of EHR data items in each subpopulation often reflect existing disparities within society, especially socioeconomic status (SES).^43^ This is because social determinants of health, such as access to health insurance and healthcare services, are typically influenced by financial income.^44^ Factors such as historical demographics, data collection procedures, and access to healthcare services can directly influence the composition of population-specific data in a healthcare system and lead to underrepresentation or overrepresentation of specific populations in the dataset. Population demographics vary across healthcare systems, resulting in distributional shifts that limit model generalizability on cohorts from different healthcare systems. In future, we will incorporate uncertainty quantification that would ensure the model expresses higher predictive uncertainty when evaluated on out-of-distribution data, for which risk estimates tend to be less reliable.

### Addressing disparities in performance across demographics groups

While differences in the performance of cancer risk prediction methods in subpopulations have been noted earlier,^3^ addressing these as part of an improved ML process is an open problem, which we have not addressed in this study. A relevant future task would be to evaluate models of this type on groups with diverse SES, perhaps by the use of residential information, such as postal zip codes, if this information is available. With sufficient population structure data, one can train a separate model for each ethnic or socioeconomic group in a particular health system. Access to such data could be obtained within a unified national healthcare system, which is not currently available in the US, or by federated learning procedures with software access to data behind institutional firewalls, which are technically feasible but difficult to negotiate. The key aim is to remove bias in the trained model(s) and achieve equal accuracy in spite of unequal risk. We anticipate that future work will achieve this aim in support of more equitable access to early detection and prevention programs.

### Stratified surveillance programs

The proposed PDAC risk model, which predicts cancer risk probabilities over various time intervals after risk assessment, offers a promising approach to differentially screen individuals based on their risk profiles. Such stratification can be either binary (at risk or not) or more granular using the real-number probabilities to stratify patients into, e.g., very-high-, high-, medium-, and low-risk groups. One can also determine thresholds in the probability values as decision parameters for various screening actions. Differential action can imply different time intervals for repeat screening visits, different procedures such as computed tomography scans, MRI scans, endoscopic ultrasound, or different advanced blood tests, e.g., for sequence variants, methylation, or fragments in cell-free DNA or for serum proteins, once these are generally accepted. Cost of the program and value of the kind of information desired would be decision parameters. Suggesting details of stratified screening programs is beyond the scope of this study. These are likely to be worked out by clinicians in consultation with developers of detection technologies, ML experts, and hospital administrators. Careful definition and prospective evaluation of stratified efficient surveillance programs is likely to lower the barrier for general acceptance in clinical practice.

### Limitations of the study

#### Medication effects

We acknowledge several limitations associated with the use of medication data in the risk prediction models. Patient adherence to prescribed medication regimens, including issues of under- or overdosing, can complicate the informational contribution of medication effects. Additionally, there is a distinction between curative medications, such as insulin for diabetes management and preventive medications like statins for cholesterol reduction or glucagon-like peptide-1 (GLP-1) receptor agonists like ozempic for weight reduction. Both may affect cancer risk by different mechanisms. Understanding these diverse impacts on disease progression is important for accurately interpreting risk profiles. This study does not look into potentially causative contributions of drugs to increase or decrease cancer risk. Investigating such relationships to establish clearer associations between medications and risk factors of cancer is of interest for future research, but prediction performance does not depend on mechanistic explanation of risk factors.

#### Comparison of the AI tool with standard clinical practice

Diagnosis trajectories can contain quasi-symptoms just prior to cancer diagnosis that are obvious risk factors and do not require ML for prediction. However, their occurrence overlaps with non-obvious risk factors, so there are no sharp boundaries. We therefore recommend using models that are trained on all or most disease states even within a few months or days prior to cancer diagnosis. This would result in the benefit of comprehensive prediction, even if some predictions of high risk based on observing quasi-symptoms (e.g., unspecified jaundice) would trivially be made by experienced clinicians. Evaluation of the innovative added benefit from the AI tool should, of course, carefully distinguish between trivial and non-trivial contributions in a patient trajectory. In reporting prediction performance, we therefore use models trained excluding the last 3 months of disease codes before cancer diagnosis (Figure 3). Larger data exclusion intervals just before cancer may also be useful. Direct comparison of prediction performance with that from standard clinical practice, however, is out of scope and would require a carefully designed controlled clinical trial.

#### Time-to-cancer diagnosis

Ideally, one would be able to predict the initiation of cancer and its growth to a detectable level. However, the AI prediction model is trained on the time point of cancer diagnosis as captured in EHRs or clinical notes, which is much later—especially for pancreatic cancer. One can perhaps improve this type of model by incorporating background knowledge of how long it typically takes for cancer to reach the stages or grades reported at first diagnosis, but this is challenging. A related question on the evaluation of prediction performance is the time interval between the time of assessment (prediction) and the time of cancer diagnosis. In the current model, we predict cancer diagnosis to occur within, e.g., 36 months, and if cancer occurs at any time point within that time frame, the prediction is scored as accurate. However, the time point of cancer diagnosis in this high-risk group in general is distributed over the 36 months, and the variation in time-to-cancer after the time point of prediction is not modeled. It may be more precisely captured—if the data are sufficiently informative—by alternative formulations of the probability model. Nonetheless, the current formulation does provide reasonable sufficient information for the design of stratified surveillance problems.

### Conclusion

The proposed AI-based risk prediction tool can be an important component of a clinical cancer management program, identifying only high-risk individuals drawn from a large, real-world population for an initial moderate-sized lower-cost targeted surveillance program. By focusing on such high-risk cohorts, one can achieve a reduction in predicted false positives and a reasonably precise identification of pre-cancerous and early-stage cases. Lowering screening costs helps ease the healthcare system’s burden, while early identification facilitates preventive measures, such as lifestyle modifications, which may delay or prevent pancreatic cancer onset. An AI-driven surveillance program—combining prediction, detection, and treatment within a clinical collaboration—provides a promising framework for equitable, effective cancer surveillance across diverse healthcare environments.

## Resource availability

### Lead contact

Requests for additional information on methods, results, and software can be directed to the lead contact, Chris Sander (sander.research@gmail.com).

### Materials availability

This study did not generate any new unique reagents.

### Data and code availability

• All the data used in the article were obtained under Institutional Review Board approval from the VA Boston Healthcare System under a waiver of informed consent. The access to VA data can be obtained by qualified investigators with necessary approvals through the VA Informatics and Computing Infrastructure (vinci@va.org).
• The codebase of our software is publicly available and can be accessed via https://github.com/thunder001/DisRiskNet and https://doi.org/10.5281/zenodo.16380009.
• Any questions on reproducibility can be directed to the lead contact.
• Any additional information required to reanalyze the data reported in this work is available from the lead contact upon request.

## Acknowledgments

This work was supported by US 10.13039/100000090CDMRP Pancreatic Cancer Risk Using Artificial Intelligence. We thank Brian Wolpin, Michael Rosenthal, Lecia Sequist, Erica Warner, and Allison Chang for their helpful discussions.

## Author contributions

Concepts, C.Z., A.K., D.R., N.R.F., and C.S.; computation, C.Z., A.K., and D.R.; supervision, D.S.M., N.R.F., and C.S.; manuscript – draft, review, and editing, A.K., C.Z., D.R., N.R.F., and C.S. By mutual agreement, joint first authors may list the article as Name et al.

## Declaration of interests

C.S. is on the SAB of Cytoreason Ltd. D.S.M. is an advisor for Dyno Therapeutics, Octant, Jura Bio, and Tectonic Therapeutic and is a cofounder of Seismic Therapeutic.

## STAR★Methods

### Key resources table

**Table:** 

REAGENT or RESOURCE	SOURCE	IDENTIFIER
**Data used for model development**		

EHR data	VA Informatics and Computing Infrastructure (VINCI). Access upon IRB approval.	VINCI@va.gov
VA Cancer Registry labels	VA Central Cancer Registry. Histology-based PDAC case definitions.	N/A
SEER Incidence Rates	Seer∗Stat software	Version 8.4.3

**Software and algorithms**		

Pytorch	N/A	https://pytorch.org
Captum	Captum	https://captum.ai/
Our code	This manuscript	https://github.com/thunder001/DisRiskNet https://doi.org/10.5281/zenodo.16380009

### Experimental model and study participant details

#### Study design

We use longitudinal ICD codes and medication data from the Department of Veterans Affairs Corporate Data Warehouse (CDW) derived in the Observational Medical Outcomes Partnership (OMOP5) format. The CDW is one of the largest databases of health data collected from VA institutions across the United States, including both inpatient and outpatient settings. Patient diagnosis is recorded in both ICD9 and ICD10 codes. We obtained diagnoses for 15,933,326 patients from 1999 to 2022 directly from the CDW as described previously.^37^ Our development cohort consists of 210,848 patients, including 18,126 PDAC cases confirmed via the VA central cancer registry and 192,722 controls without any PDAC diagnosis codes. A separate hold-out set of 987,693 patients (1,300 PDAC; 986,393 controls) was reserved for retrospective performance evaluation.

All analyses were done under a VA Boston Healthcare System IRB-approval on a VA compute server. Data handling complied with the VA policy.

### Method details

#### Data source - US VA Corporate data Warehouse

We use longitudinal ICD codes and medication data from the Department of Veterans Affairs Corporate Data Warehouse (CDW) derived in The Observational Medical Outcomes Partnership (OMOP5) format. The CDW is one of the largest databases of health data collected from VA institutions across the United States, including both inpatient and outpatient settings. Patient diagnosis is recorded in both ICD9 and ICD10 codes. We obtained diagnoses for 15,933,326 patients from 1999 to 2022 directly from the CDW as described previously.^31^

The medication data in CDW is challenging to use directly because it is dispersed across multiple tables, which requires data extraction and integration efforts to obtain medication histories of patients. To overcome this complexity, we obtained medication usage information by leveraging the advantage of an OMOP (version 5) database, which is derived from CDW and has data processed for research use.

In brief, the *drug_exposure* table in OMOP was constructed by mapping and aggregating multiple raw medication tables from CDW. Subsequently, a standardized algorithm developed by the observational health data sciences and informatics (OHDSI) community was used to process medication information from the *drug_exposure* table to form the *drug_era* table. The *drug_era* table contains continuous intervals for each drug, representing periods during which a patient is assumed to be exposed to the drug, categorized under the concept class “*Ingredient*” in the OMOP common data model. We extracted all medication usage information for each patient from the *drug_era* table.

To ensure relevance and accuracy of the medication data used for training the model, we included only active medications. Inactive ingredients were filtered out using the FDA’s inactive ingredient information.^45^ The following sections describe in detail how we constructed the training and evaluation datasets (Figure 1S).

#### Filtering data against US VA cancer registry

Due to the issue of upcoding of diagnosis codes discussed in the introduction, the ICD codes for patients often do not accurately reflect their actual health conditions. This discrepancy complicates the identification of true pancreatic cancer cases; simply relying on the presence of ICD codes denoting pancreatic cancer would result in a high number of false positive labels (nearly 50%, data not shown). To remedy this issue, we retrieve a list of verified pancreatic cancer patients from the VA Cancer Registry, a centralized repository of cancer patients data within the VA system. Inclusion of patients in the registry is governed by stringent criteria and verification steps that extend beyond individual ICD codes.

Using a set of verified positive cases from the cancer registry, we filter out our dataset by excluding any patients who had ICD codes indicating pancreatic cancer but did not appear in the cancer registry. Conversely, we also excluded patients listed in the cancer registry who lacked corresponding ICD codes in the CDW EHR, although such instances were rare. The filtering step enhances the reliability and accuracy of pancreatic cancer labels in our dataset.

Pancreatic cancer cases in the VA cancer registry are identified via histology codes (Table S1) and exclude non-PDAC tumors. Specifically, we follow the histology-code algorithm of,^46^ including only highly malignant PDAC cases and omitting less aggressive subtypes (e.g., neuroendocrine neoplasms). Table S1 lists primary site codes, corresponding to ICD-*O*-3 morphology codes, and histologic descriptions that defined our final PDAC cohort.

#### Dataset preparation for model development and evaluation

We use the US-VA database to construct training and evaluation datasets, based on a total of 15,926,415 patients, including 19,426 cases of pancreatic cancer. Overall data statistics including data splits used for model development and evaluation are in Table 1. We use each patient’s time-based trajectory of International Classification of Diseases (ICD) codes for diagnoses and active ingredients from prescription medications. Consistent with the approach in earlier work,^31^ we restrict ICD codes to three-character categories within the ICD hierarchy to focus on fairly broad diagnostic groups. Timestamps associated with the diagnosis or medication event codes are incorporated in the model as positional encodings to account for the temporal aspect of patients’ clinical histories.

To train the AI model, we subsample trajectories of fixed lengths for each patient, starting at the first recorded event and ending at various endpoints. This subsampling approach ensures a more comprehensive modeling of patient risk, as it captures periods when specific symptoms develop. As a result, the same patient can have trajectories representing variations in cancer risk over time. These variations can occur for a number of reasons such as changes in lifestyle, disease states or the use of specific medications. This approach allows us to interpret each trajectory as a realistic and dynamic representation of a patient’s health over time.

The following sections describe in detail how we constructed the training and evaluation datasets (Figure 1S).

#### Filtering data against US VA cancer registry

Due to the issue of upcoding of diagnosis codes discussed in the introduction, the ICD codes for patients often do not accurately reflect their actual health conditions. This discrepancy complicates the identification of true pancreatic cancer cases; simply relying on the presence of ICD codes denoting pancreatic cancer would result in a high number of false positive labels (nearly 50%, data not shown). To remedy this issue, we retrieve a list of verified pancreatic cancer patients from the VA Cancer Registry, a centralized repository of cancer patients data within the VA system. Inclusion of patients in the registry is governed by stringent criteria and verification steps that extend beyond individual ICD codes.

Using a set of verified positive cases from the cancer registry, we filter out our dataset by excluding any patients who had ICD codes indicating pancreatic cancer but did not appear in the cancer registry. Conversely, we also excluded patients listed in the cancer registry who lacked corresponding ICD codes in the CDW EHR, although such instances were rare. The filtering step enhances the reliability and accuracy of pancreatic cancer labels in our dataset.

#### Construction of a development and a retrospective evaluation set

To construct our training and evaluation datasets, we first extracted ∼10 million patient records from the CDW. We hold-out ∼1 million of these patients to form a retrospective evaluation set. From the remainder, we include all verified positive pancreatic cancer patients and a subset of negative patients, resulting in a development set totaling 210,848 patients. This approach reduces the class imbalance between positive and negative cases in the training process, which improves the model’s ability to learn effectively from both classes – particularly from a diverse set of positive cancer histories, while managing computational resources. This set is used during model development, and a retrospective evaluation set is used to assess performance after model development is completed.

#### Data preprocessing

We use trajectory sampling to increase the number and diversity of patient history sequences used during training. Trajectory sampling involves sampling multiple subsequences from patients’ histories, providing a larger and more varied set of inputs for the model. Specifically, for each patient, we randomly select an event within their sequence of ICD codes and medication records. We then use the patient’s history up to that event as an input data and predict the risk from that event forward. We refer to these sampled subsequences as patient ‘trajectories’. This approach allows the model to learn from different stages of a patient’s medical history, capturing a wider range of health conditions.

For patients with pancreatic cancer, trajectories are sampled from time intervals before the cancer diagnosis. For patients without pancreatic cancer, trajectories are sampled from at least two years before the end of their record to minimize cases where a patient developed pancreatic cancer shortly after the prediction timepoint, which might not be recorded in their EHR history, e.g., if they leave the health care system. This sampling procedure means that positive cancer patients can produce trajectories with negative labels. For e.g., if a patient’s history is taken up to an event in the fifth year of their record, but they do not develop cancer until the fifteenth recorded year (past the largest predicted time interval of our model). Such trajectories provide challenging negative samples, as ideally the model should learn not only that someone may get cancer, but also predict a specific time interval in which this could occur.The sampling process further addresses the imbalance caused by low incidence of pancreatic cancer. The purpose of the test set is to evaluate the model’s performance on data with a data distribution similar to the training set. During training, four random trajectories are sampled for each patient in each epoch. The distribution of trajectories in different prediction intervals is shown in Figure S2.

Additionally, a separate hold-out set is used to assess the model’s performance on data that more closely reflects a realistic setting. During training, four random trajectories are sampled for each patient in each epoch. At evaluation time, rather than random sampling, we take the last four trajectories before either the time of cancer diagnosis or two years before the end of the record (depending on whether the patient has cancer). This ensures that different models are evaluated on a consistent set of trajectories, using the most information available in the patient records.

#### Risk-prediction model development

This section describes the ML framework that uses the longitudinal nature of EHRs for PDAC risk prediction. The input to the model consists of contiguous subsequences of diagnosis and prescription codes, along with their corresponding timestamps, extracted from a patient’s medical record. These subsequences enable the model to assess a patient’s evolving cancer risk over a series of time points leading up to a potential cancer diagnosis.

##### Encoder

The first step in the model’s architecture is to transform each event (i.e., one diagnostic or medication code) in a trajectory into a continuous feature representation, achieved using a two-step process. First, each event is mapped to a low-dimensional continuous feature vector using an embedding lookup table (learned during training). Next, positional encodings are added to the embeddings to account for the temporal order of events within a trajectory. These positional encodings are derived from the timestamps following (Placido et al., 2023) and added to the embeddings of respective events. Additionally, patient age, a known risk factor for pancreatic cancer, is also encoded in a similar manner. This encoding allows the model to handle irregularly spaced events within trajectories. In this way, a trajectory of length N is mapped to a feature matrix $X\in R^{N\times d_{in}}$, where d is the dimensionality of the latent space.

##### Positional encodings

To incorporate temporal information, we apply a time encoding method based on the timestamps associated with the sequence events. This approach is particularly useful for capturing the temporal dynamics of medical events and aids in handling trajectories with irregular intervals. The first step involves generating positional time embeddings P, using a set of multipliers, defined as follows,

$$M=\frac{2\;\pi }{linspace\left(start,end,d_{in}\right)}\in R^{d_{in}}$$

where start and end denote the minimum and maximum values for the embedding range, linspace generates linearly spaced $d_{in}$ points between start and end. Given the time deltas Δt, for each event in the sequence, the positional embeddings are computed using the cosine function as, $P=\cos\left(\Delta t\;\cdot \;M\right)\in R^{N\times d_{in}}$ . The embeddings P are then integrated with the original feature matrix X by a combination of two transformations: $E_{scale}$ that scales the original features, and $E_{add}$ that analogous to a bias is added to features. Both transformations are derived from P using linear transformations $W_{scale},W_{bias}\in R^{d_{in}\times d_{in}}$ and,

$$E_{scale}=PW_{scale}{,E}_{add}=PW_{add}$$

The final time-informed feature embedding is computed as:

$${X=E}_{scale}⊙X+E_{add}$$

where ⊙ denotes the element-wise multiplication.

##### Transformer neural network

Next, we use a Transformer architecture to capture sequential interdependencies between events within a trajectory. The Transformer uses a self-attention mechanism, enabling it to learn pairwise relationships between all events in the input trajectory.^47^ The Transformer’s output is then aggregated over the entire trajectory length, resulting in a fixed-size latent representation that conceptually encapsulates the patient’s health state at a specific time interval. Formally, consider Q, K, and V be the query, key, and value matrices, respectively, obtained by linear projections of the input X,

$$Q=X{W_{Q}}^{T},K=X{W_{K}}^{T},V=X{W_{V}}^{T}$$

where $W_{Q},W_{K},W_{V}\in R^{d\times d_{in}}$, and d is the dimensionality of a projection space. The attention mechanism is then defined as,

$$A=\frac{QK^{T}}{\sqrt{d}},Z=\sigma \left(A\right)V$$

here A is a self-attention matrix, σ is softmax along the column, and Z is the intermediate representation of an input trajectory. It is typical to use a Transformer with multiple heads that increase its capacity to model intricate relationships. The multi-headed attention mechanism combines multiple attention heads to capture different types of dependencies:

$$Z_{agg}=\left[Z_{1}\cdots Z_{H}\right]\;W_{agg}$$

where, [⋯] is a concatenation along the columns, $W_{agg}\in R^{Hd\times d}$ is a learnable weight matrix that combines the outputs of the attention heads, and $Z_{agg}$ is the latent representation aggregated across all heads. The output of the multi-headed attention mechanism is passed through a feedforward layer, layer normalization, a linear layer, and a pooling layer to obtain the fixed length encoding $z\in R^{d}$ of an input trajectory.

##### Risk regression model

In the final step, the fixed-length latent representation z serves as covariates in a risk prediction model. Specifically, a regression model includes parameters $\beta \in R^{d\times 5}\text{,}$ bias $b\in R^{d\times 1}$ that predicts the risk within five pre-defined time intervals (e.g., 0–3, 3–6, 6–12, 12–36, 36–60 months),

$$\lambda \left(t|z\right)=ReLU\left(z^{T}\beta +b\right)$$

where ReLU(x)=max(0,x) stands for rectified-linear unit. The cumulative risk of cancer occurrence for time intervals, such as, 0–3, 0–6, 0–12, 0–36, 0–60 months, can then be obtained as,

$$s_{T}\left(z\right)=s\left(T\geq t\;|\;z\right)=\lambda \left(z\right)+\sum_{t}^{T}\lambda \left(t|z\right)$$

where $\lambda \left(z\right)=z^{T}c$ is a baseline risk of an input trajectory parametrized through c $\in R^{d\times 1}$; the cumulative probability of cancer in the respective time interval is obtained as,

$$p_{T}\left(z\right)=sigmoid\left(s_{T}\left(z\right)\right)\text{.}$$

For the five selected intervals, the respective probabilities $\left\{p_{1},p_{2},p_{3},p_{4}{,p}_{5}\right\}$ are optimized using binary cross entropy loss against a target sequence $\left\{y_{1},y_{2},y_{3},y_{4}{,y}_{5}\right\}$. The parameters of the risk model, embedding layer, and Transformer layer together referred as θ are learned jointly by optimizing the combined binary cross-entropy loss over the five chosen time intervals.

$$loss=\frac{1}{N}\frac{1}{N_{T}}\;\sum_{i,t}^{{N\;N}_{T}}\;\left[y_{i,t\;}\;\log\;\left(p_{t}\left(z_{i}\right)\right)+\left(1-y_{i,t\;}\right)\;\log\;\left(1-p_{t}\left(z_{i}\right)\right)\right]+\lambda_{2\;}|\left|\theta \right|{|}_{2}$$

#### Training and evaluation setup

The development dataset of VA subjects described in Table 1 was split into training, development, and test datasets according to an 80:10:10 split. This test set simply gives us an estimate of performance on data with an inflated number of positive cases. This is a useful sanity check after model training, but not strictly necessary as our final reported performance is based on a held-out evaluation set. Transformer models were trained using the training dataset, the best performing model was chosen using development datasets. Due to the low incidence of pancreatic cancer patients, balanced sampling of the trajectories of patients in the training set was used to obtain approximately equal numbers of trajectories from cancer and non-cancer patients. The binary cross-entropy loss function was used for model training with an initial learning rate of 0.001 and trained for 20 epochs. During training, if the validation AUROC failed to improve for more than four epochs, the model with the best validation AUROC up to that point was reloaded, the learning rate was halved, and training then continued. The final model saved after training was the one with the highest validation AUROC across all training epochs. All models were trained on an NVIDIA A100 GPU with 40GB of GPU memory. The average training time for the model was 12 h, for evaluation 10 h, and for attribution 6 h.

##### Model architecture and hyperparameter setup

We use a Transformer architecture as an encoder neural network in our model. Specifically, the encoder consists of *‘L’* Transformer layers, each using a multi-head self-attention (MHSA) mechanism with ‘*H’* heads. Each has a dimensionality of *‘d’* for the key, query and value transformations. The output of the MHSA module is combined with the original input through a residual connection and then normalized using a layer normalization to improve training stability. This is followed by a position-wise feedforward network consisting of two fully connected layers of dimensionality ‘d’ with ReLU activation functions. The output of the feedforward network is combined with its input via a second residual connection and layer normalization.

For risk regression, we implement a time-to-event probability transformation. This transformation includes two fully connected linear layers: one maps the input features (dimensionality ‘d’) to hazard rates across the five intervals, and the other computes a baseline hazard term. The hazard outputs are passed through ReLU activation to ensure they are non-negative and are combined with the baseline hazard to output the risk probability for five intervals (as discussed above).

We conducted a grid search over the following configurations of hyperparameters in the encoder network L = {1,2,4}, H = {4,8,16}, d = {64, 128}. Additionally, we also included different values of learning rate lr = {0.0005, 0.001, 0.005, 0.01}, weight decay = {0, 0.01, 0.1, 0.5} in the Adam optimizer and dropout probability = {0, 0.1, 0.2, 0.5}. The optimal hyperparameters were determined to be L = 1, H = 16, d = 64, lr = 0.001, and without dropout. We trained all models for a maximum of 100 epochs, monitoring convergence every five epochs.

#### Identifying clinically relevant codes using attribution analysis

To highlight the most predictive features to our model, we use the Integrated Gradients (IG) (Sundarajan et al., 2017) method to compute feature attributions. IG provides estimates of the contributions of each individual feature in an input to the model output. To do so, IG computes a path integral over the gradients of the model output with respect to inputs. The integration path starts from an uninformative baseline input and ends at the input for which we are computing the attributions. Formally, this can be written as

$$IG_{i}\left(F,x,x^{base}\right)=\left(x_{i}-{x_{i}}^{base}\right)\times \int_{\alpha =0}^{1}\frac{\delta F\left(x^{base}+\alpha \left(x-x^{base}\right)\right)}{\delta x_{i}}d\alpha$$

where $x,x^{base}$ and F are the input, baseline and model, respectively. ${IG}_{i}$ is a local attribution method, and only computes the feature attributions for a single input.

To estimate the model’s reliance on particular features across many examples, we compute the fraction of the time an ICD code or medication occurs in the top 10% highest attributed features in an input sequence. This approach allows us to identify diagnoses or medications that are important to model predictions. Relevant features should consistently rank highly across many inputs, while less important features should appear in the top rankings less frequently. By computing the fraction of the time a code is highly ranked and normalizing this by its overall occurrence, we ensure that frequently occurring codes are not deemed important solely due to their high frequency. In summary, this attribution approach highlights the global importance of codes while accounting for contextual relevance across trajectories.

We apply IG with the above aggregation scheme to the 1000 highest and lowest predicted risk trajectories in our training data. This approach targets confident model predictions, avoiding less informative low or mid risk features. True positive cases that have only a moderate level of predicted risk would also likely have uninformative attributions, since those attributions are a function of the model. Taking 1000 of those with the highest predicted risk means that the true positives are also predicted to be very likely positive, and therefore we would expect the attributions to look reasonable.

#### Baseline models

We compare our proposed model with two baseline methods: an MLP and LR. The LR model uses a ‘bag-of-events’ representation of a patient’s ICD and medication history as input and produces five monotonically increasing risk scores using the risk regression model as discussed above. This representation is obtained by transforming the sequence of ICD and medication codes into a vector, where each dimension corresponds to the frequency of a specific code normalized by the total number of codes in the trajectory. Although this encoding captures information about the occurrence of codes, it ignores their temporal ordering. In contrast, the MLP uses the same input embeddings as our Transformer model but processes each embedding independently through two fully connected layers, instead of using self-attention to learn interdependencies among the codes. The output of the MLP is obtained by averaging over tokens and passed through the same risk regression head used in the Transformer model to produce the five output risk scores. The sequential model, which uses a Transformer architecture, achieves the highest AUROC scores (Figure S3), supporting the hypothesis that a temporal model of diagnostic and medication data is a more effective approach for cancer risk prediction.

### Quantification and statistical analysis

Evaluation of each model was done using a retrospective set described in the second part of Table 1. The AUROC score was used for evaluation of overall model performance and the 95% confidence interval was computed using 100 resampling of bootstrapping with replacement. We further report the incidence ratio (IR), positive predictive value (PPV) and standardized incidence ratio (SIR) for sets of highest-risk patients to quantify the benefit of our model in a realistic application of surveillance programs.

**Standardized Incidence Ratio (SIR)** is a score used to assess how well a risk prediction model can identify high-risk patients for a disease, such as PDAC. It compares the observed positive cases in a high-risk cohort to the expected baseline incidence. The expected baseline incidence in high-risk patients is calculated by summing the age-group-specific incidence rates from the standardized nationwide database, weighted by the number of patients in each age group, race, and sex combination present in the database.

Denote AgeGroups={50−54,55−59,⋯,85+} as the set of age intervals used for risk stratification, Race={Black,Whites,Others(Asian,Hispanic)} as the set of racial groups, and Gender={Male,Female}. Let H be the set of high-risk patients (age ≥ 50), $f^{x,y,s}$ be the number of patients with age x, race y, sex s in the database, $TP_{x,y,s}$ be a count of true positives within that group, and Incidence(x,y,s) be the baseline PDAC incidence rate (per year) that can be obtained from the US-VA or the SEER database for the corresponding age, race, and sex stratum (Table S2). SIR is then defined as,

$$SIR=\frac{Observed\;Positive\;Cases\;in\;H}{Expected\;baseline\;Incidence\;in\;H}=\frac{\sum_{x\in AgeGroup\;}\sum_{y\in Race\;}\sum_{s\in Sex\;}TP_{x,y,s}}{\sum_{x\in AgeGroup\;}\sum_{y\in Race\;}\sum_{s\in Sex\;}\sum_{t\in T}Incidence\;\left(x+t,y,s\right)\times f^{x,y,s}\;}$$

where T = {0, 1, 2, 3} is the time-window of cancer-occurence in units of years. For values of t less than 1 year, we round down to the previous year when querying the database. For example, when computing SIR for a patient at the 6 or 12 months timepoints there is no summation over T.

The 95% confidence interval of SIR is computed using the formula below,^48^

$$95\%\;CI={\left(\sqrt{N_{Observed}\;}\pm \;0.98\right)}^{2}\;/\;N_{Expected}$$

$$N_{Observed\;}=\text{Observed}\;\text{positive}\;\;\text{cases}\;\text{in}\;\text{high}\;\text{risk}\;\text{cohort}\;H\text{.}$$

$$N_{Expected}=\text{Expected}\;\text{baseline}\;\text{incidence}\;\text{in}\;\text{high}\;\text{risk}\;\text{cohort}\;H\text{.}$$

A SIR value greater than 1 indicates that the risk prediction model has identified a cohort with a higher-than-expected incidence rate than the baseline population, suggesting that the model effectively identifies high-risk individuals who are more likely to develop PDAC. Conversely, a SIR value less than 1 implies that the model is not accurately identifying the high-risk cohort. The SIR metric provides a standardized way to evaluate the performance of a risk prediction model in identifying high-risk patients, accounting for differences in age, race, and sex distributions between the study population and the baseline population. It is particularly useful in PDAC, where early detection and risk stratification are crucial for improving patient outcomes.

**Incidence Ratio (IR)** is defined as the ratio of the precision of the prediction model to the baseline incidence rate obtained from the population,

$$IR=\frac{PPV}{Incidence}$$

where Incidence refers to the baseline PDAC incidence rate also determined from the US-VA dataset and PPV stands for positive predictive value. The key difference between IR and SIR is that SIR adjusts for demographic factors such as age, race, and gender, while the IR does not account for these variables. Placido et al., 2023 refers to IR as relative risk (RR).

**Positive Predictive Value (PPV)** measures the proportion of true positive predictions among all positive predictions made by the model. Formally,

$$PPV=\frac{True\;Positives\;\left(TP\right)}{True\;Positives\;\left(TP\right)+False\;Positives\;\left(FP\right)}$$

where TP stands for true positives and FP for false positives. A higher value means the model has a better rate of TPs relative to FPs. If the model predicts a positive outcome, PPV measures how probable it is that the prediction is correct.

## Supplemental information

Document S1. Figures S1–S8 and Tables S1 and S2

Document S2. Article plus supplemental information
