# Supplementary material for: Pancreatic cancer risk prediction using deep sequential modeling of longitudinal diagnostic and medication records
Source: Cell Rep Med. 2025 Sep 16;6(9):102359. doi: 10.1016/j.xcrm.2025.102359 (PMC12490214; doi:10.1016/j.xcrm.2025.102359)
Supplement: Document S1. Figures S1–S8 and Tables S1 and S2 [file mmc1.pdf]

**Cell Reports Medicine, Volume *6***

## Supplemental information

# Pancreatic cancer risk prediction using deep sequential modeling of longitudinal diagnostic and medication records

**Chunlei Zheng, Asif Khan, Daniel Ritter, Debora S. Marks, Nhan V. Do, Nathanael R. Fillmore, and Chris Sander**

## Supplementary Material

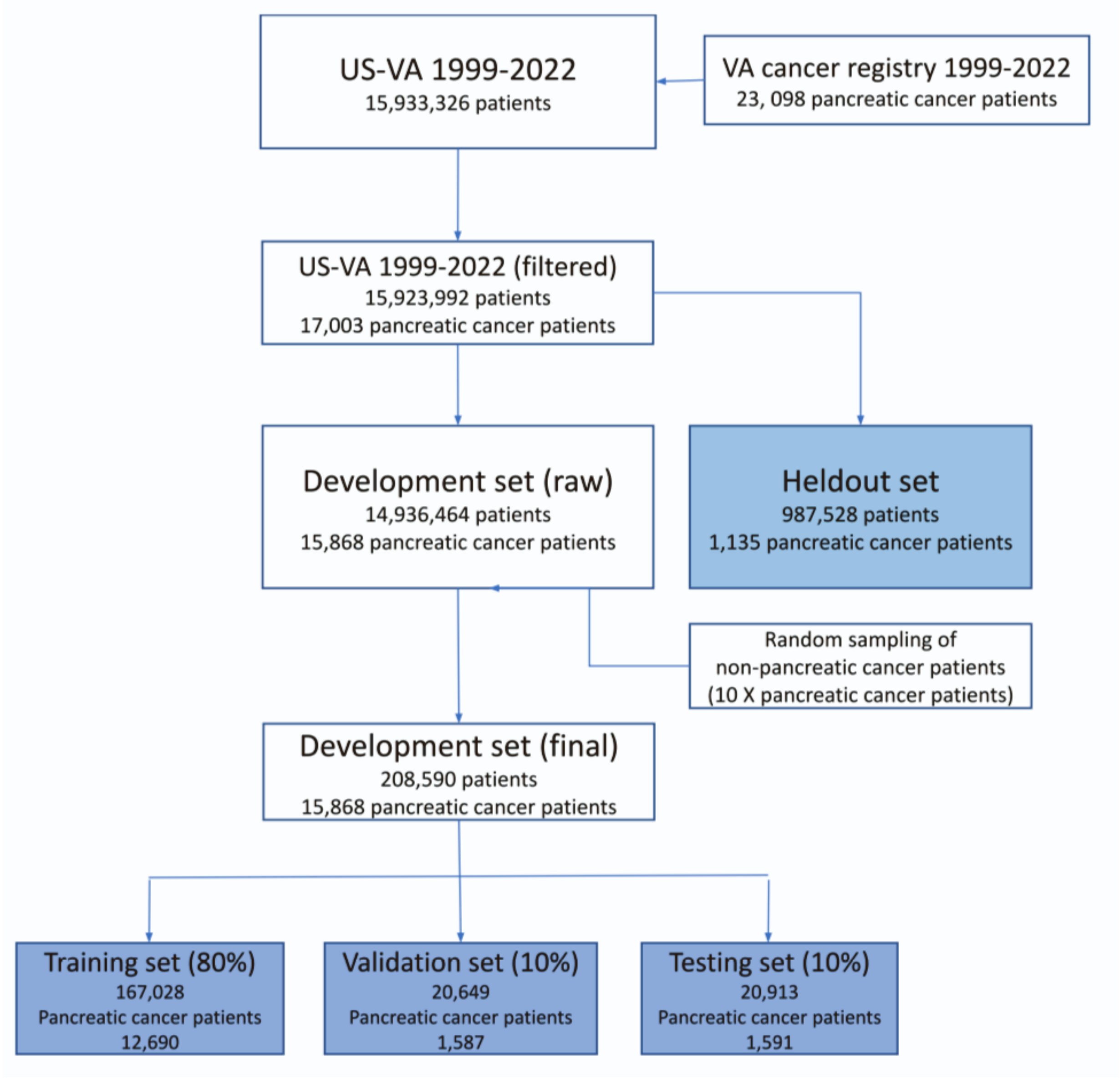


***Figure S1. (Related to star methods)*** *Flowchart of data extraction and preparation for training ML models.*

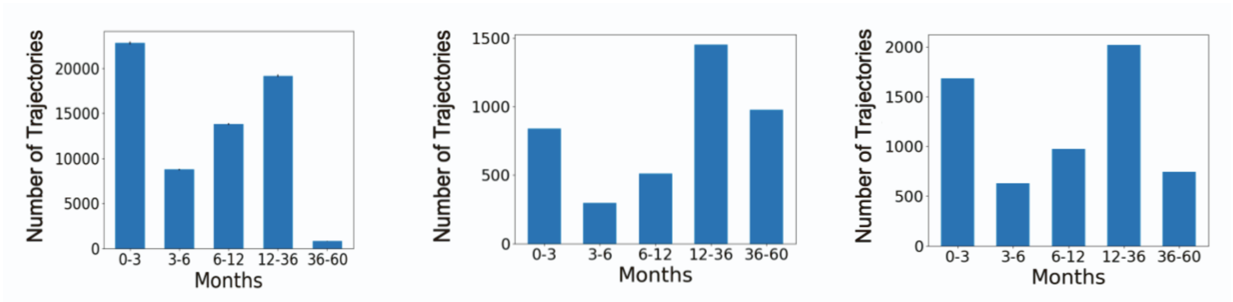


***Figure S2.*** ***(Related to star methods)*** *Observed occurrence of cancer following the prediction point for (A) the training set, (B) the validation set, and (C) the test set.*

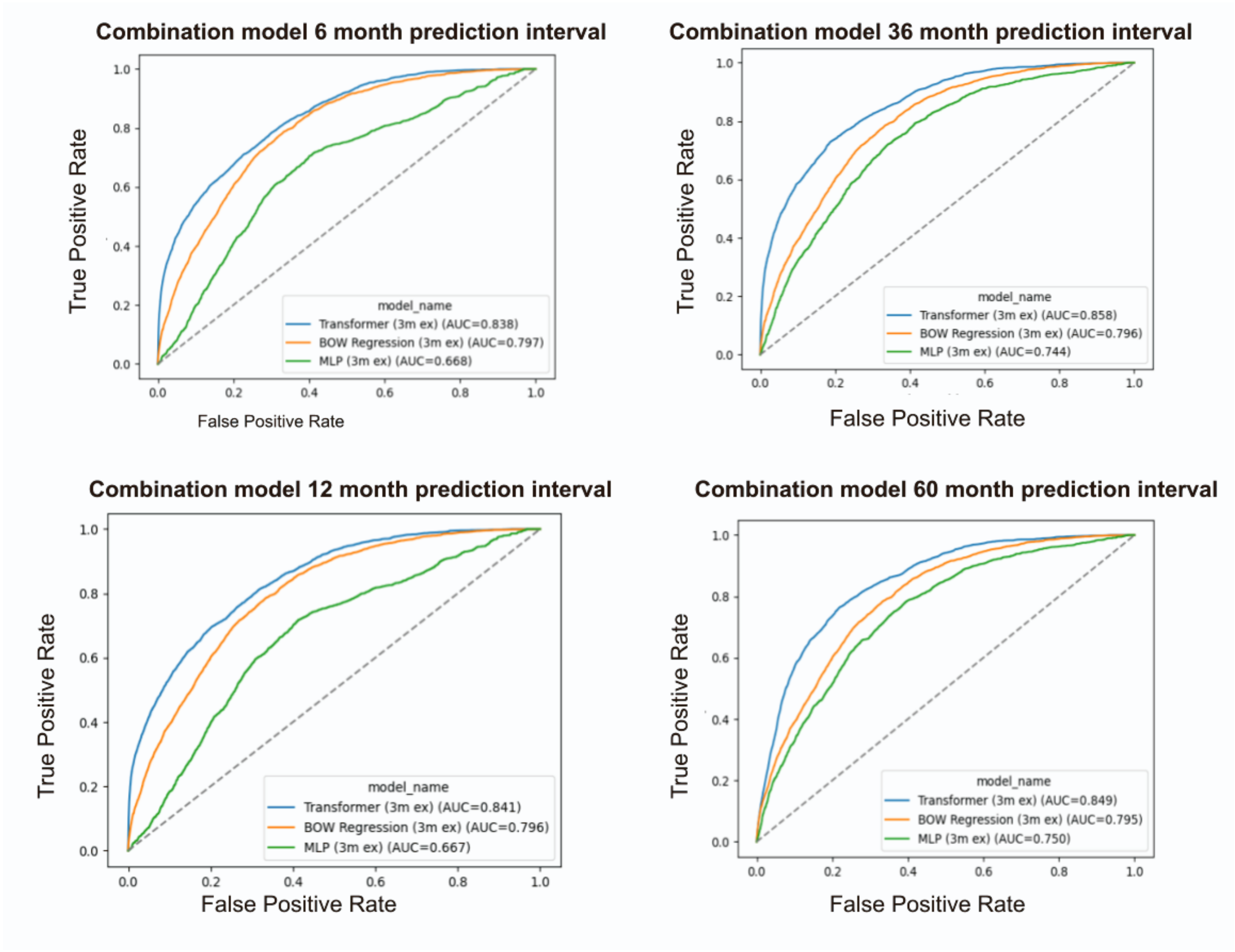


***Figure S3.*** ***(Related to Figure 3) Comparison of sequential models with baselines on a test dataset.*** *All baselines are trained on combined (medication and diagnosis) dataset. Each plot compares AUROC scores of the Transformer (sequential) model with two non-sequential baselines, the logistic regression (BOW = bag of words) and MLP for the 6, 12, 36, and 60 months prediction windows.*

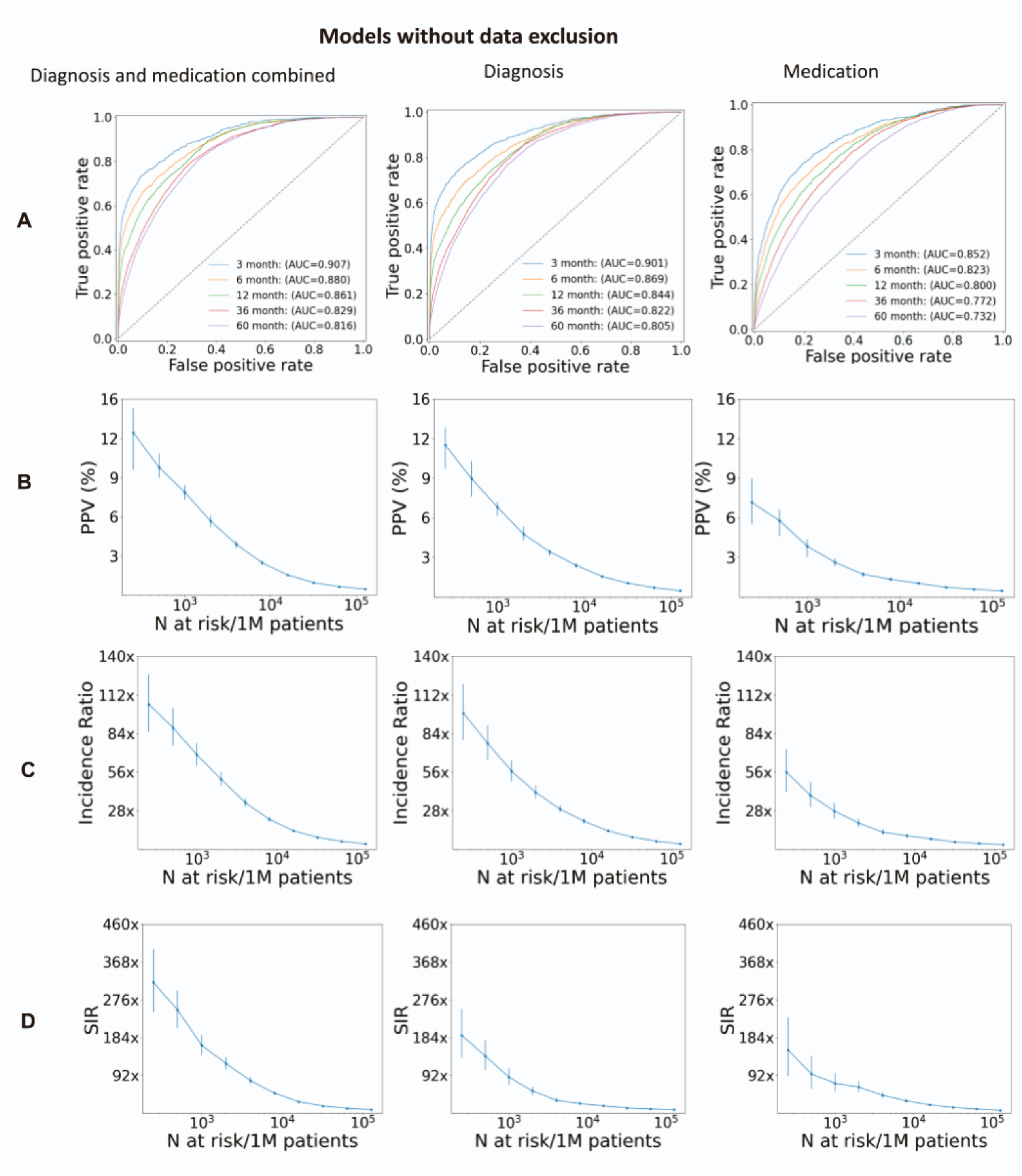


***Figure S4. (Related to Figure 3) Comparison of the performance of models trained without a data exclusion window: diagnostic data only, medication data only, and combination of both.*** *Left panels: combination of diagnosis and medication data. Middle panels: diagnosis data only. Right panel: medication data only. (A) AUROCs for different prediction time intervals, (B) Positive predictive value (PPV) for high-risk groups per 1 million patients, (C) Incidence ratio (IR) of high-risk groups per 1 million patients, and (D) Standardized incidence ratio (SIR) of high-risk groups per 1 million patients. The prediction performance improved compared to the three month exclusion, likely due to the inclusion of quasi-symptoms that appear before an actual diagnosis. These results demonstrate the AI tool can capture features that a well-trained clinician might identify.*

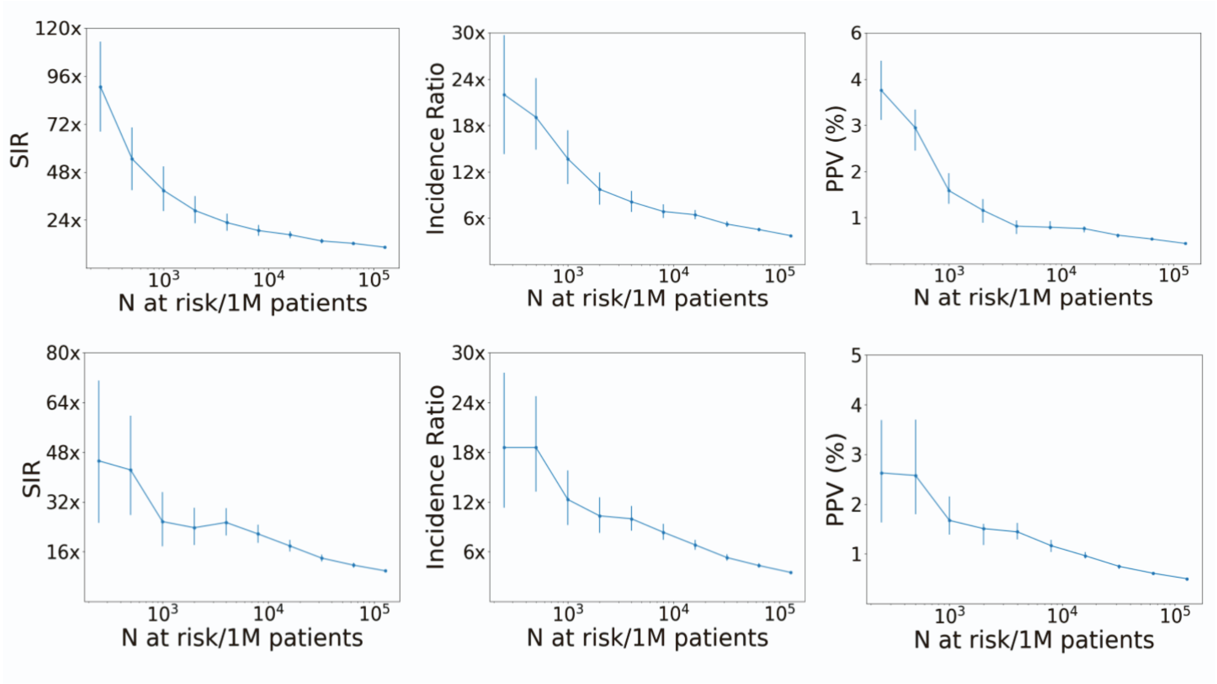


***Figure S5. (Related to Figure 3)*** *Evaluation of a 36-month prediction window model trained using data with data exclusion.* ***Top row:*** *results for a model trained with a six month exclusion window.* ***Bottom row:*** *results for a model trained with a twelve month exclusion window. For the longer data exclusion windows, as expected, the prediction performance decreases.*

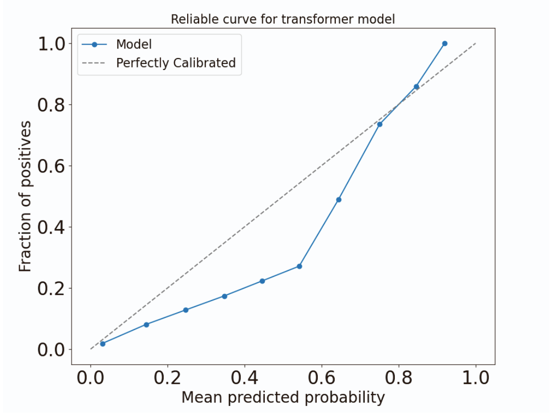


***Figure S6. (Related to Figure 2) Calibration curve of a 36-month prediction interval model.*** *To assess the agreement between predicted and observed risks, we evaluated the model's calibration and plotted the calibration curve for the 36-month prediction horizon. Figure 6S shows probabilities align closely with actual event rates, indicating the model is calibrated even with low absolute occurrence of pancreatic cancer.*

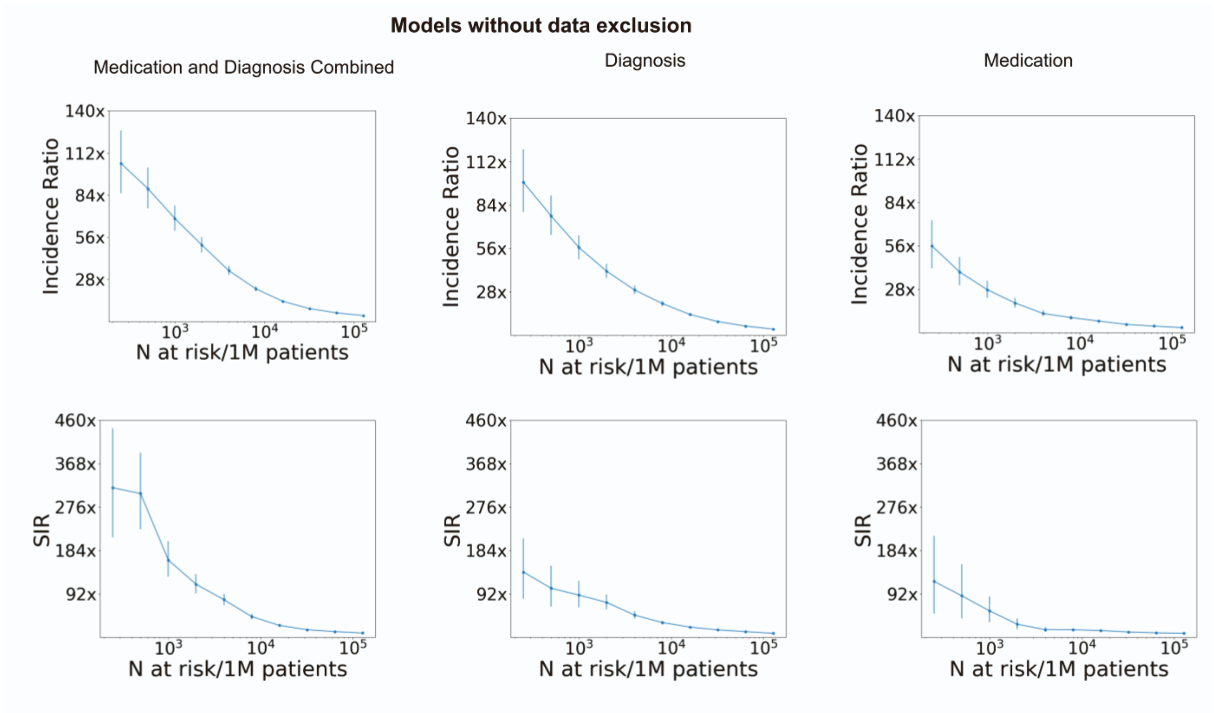


***Figure S7. (Related to Figure 2) Comparison of Incidence Ratio and SIR of models trained without a data exclusion window across three data types, using SEER incidence rate: diagnostic data only, medication data only, and combination of both.*** *Left panels: combination of diagnosis and medication data. Middle panels: diagnosis data only. Right panel: medication data only.*

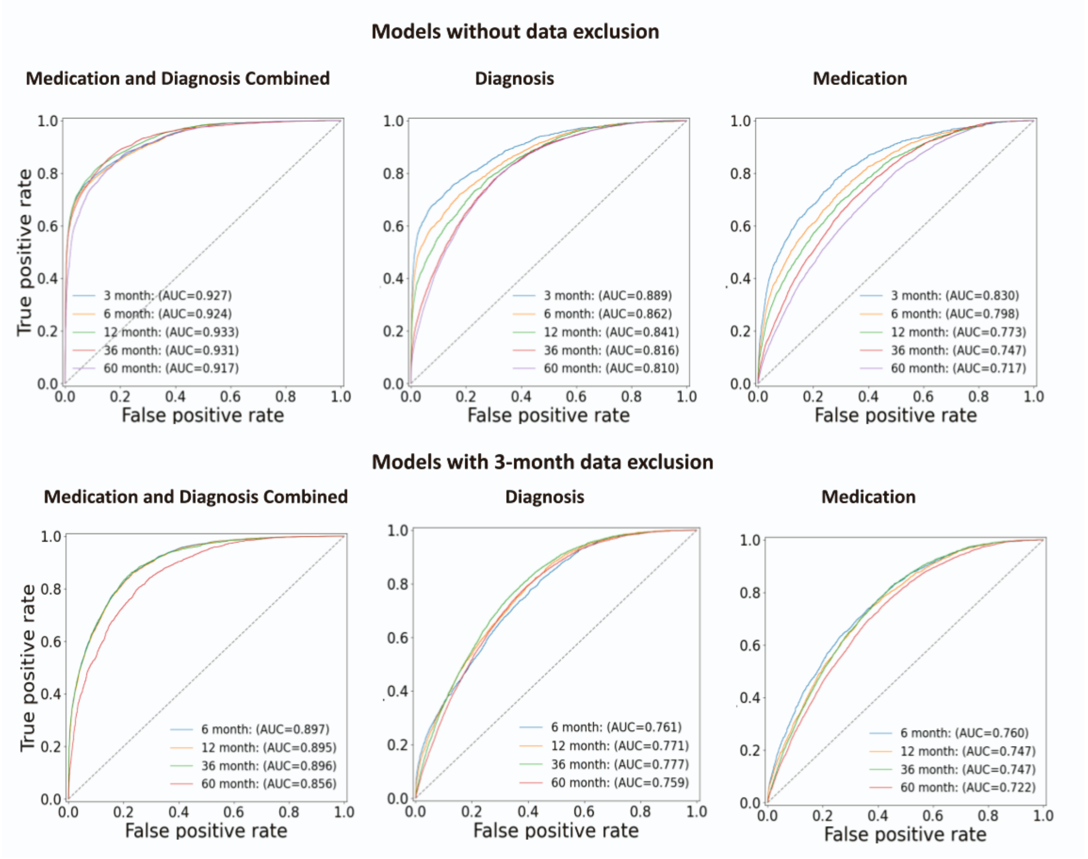

***Figure S8.*** *Evaluation on a held-out test set comprising 10% of the data reported in Table 1A, with a higher proportion of PDAC cases compared to the test data reported in Table 1B. These results reflect the model's performance on a test set that mirrors the cancer-to-non-cancer ratio present in the training set.*

**Table S1.** ***(Related to star methods)*** *Criterion for inclusion of pancreatic cancer patients. Primary sites, their description and icd 10 codes used for constructing cohorts of pancreatic cancer patients. Controls (non-pancreatic cancer) are cases that are not in the cancer registry as pancreatic cancer and do not have ICD codes for any pancreatic cancer.*

| PrimarySiteIEN | Description | ICD 10 |
|---|---|---|
| 67250 | PANCREAS, HEAD | C25.0 |
| 67251 | PANCREAS, BODY | C25.1 |
| 67252 | PANCREAS, TAIL | C25.2 |
| 67253 | PANCREAS, DUCT | C25.3 |
| 67257 | PANCREAS, OTHER | C25.7 |
| 67258 | PANCREAS OVERLAP | C25.8 |
| 67259 | PANCREAS NOS | C25.9 |

The cohort includes all pancreatic cancer patients with primary site codes in above table, excluding those with histology codes listed below:

<table>
<tr><th>Histology ICDo3</th><th colspan="2">Description</th></tr>
<tr><td>8000</td><td colspan="2">Neoplasm, malignant</td></tr>
<tr><td>8001</td><td colspan="2">Tumor cells, malignant</td></tr>
<tr><td>8150</td><td>Papillary carcinoma</td><td rowspan="10">Endocrinomas</td></tr>
<tr><td>8151</td><td>Verrucous carcinoma</td></tr>
<tr><td>8152</td><td>Glucagonoma, malignant</td></tr>
<tr><td>8153</td><td>Gastrinoma, malignant</td></tr>
<tr><td>8154</td><td>Mixed pancreatic endocrine and exocrine tumor, malignant</td></tr>
<tr><td>8155</td><td>Vipoma</td></tr>
<tr><td>8156</td><td>Somatostatinoma, malignant</td></tr>
<tr><td>8157</td><td>Enteroglucagonoma, malignant</td></tr>
<tr><td>8158</td><td>ACTH-producing tumor</td></tr>
<tr><td>8240-8249</td><td colspan="2">Carcinoid tumor, malignant</td></tr>
<tr><td>9140</td><td colspan="2">Kaposi sarcoma</td></tr>
<tr><td>9590-9992</td><td colspan="2">Lymphoma, plasma cell tumors, mast cell tumors, etc.</td></tr>
</table>

***Table S2 (Related to Star Methods). Baseline incidence rate stratified by age, and race in US-VA and SEER database.*** *The SEER incidence were obtained using SEER*Stat software version 8.4.3*[1]*. The pancreatic cancer incidences in US-VA were computed using* ***Incidence rate = (New cancers / Population)*** *with subsampling described in data source.* For each sampled trajectory, the age, sex, race, and its pancreatic cancer status within 12 months were computed, the incidence of pancreatic cancer is defined by the ratio of the number of trajectories with pancreatic cancer to the total number of trajectories a.k.a. population.

| Age Group | Race (White, Black) | Sex | SEER Incidence | US-VA Incidence |
|---|---|---|---|---|
| **50-54** | black | Female | 0.00013338 | 0.00036411 |
| **50-54** | black | Male | 0.00019127 | 8.512E-05 |
| **50-54** | white | Female | 7.779E-05 | 0.00020017 |
| **50-54** | White | Male | 0.00012217 | 0.00028827 |
| **55-59** | black | Female | 0.00023298 | 6.122E-05 |
| **55-59** | black | Male | 0.00033398 | 0.00081471 |
| **55-59** | white | Female | 0.00014521 | 0.00011076 |
| **55-59** | White | Male | 0.00022726 | 0.00049353 |
| **60-64** | black | Female | 0.00036045 | 0.00018398 |
| **60-64** | black | Male | 0.00051581 | 0.00065415 |
| **60-64** | white | Female | 0.00022726 | 0.00026773 |
| **60-64** | White | Male | 0.00033968 | 0.00069301 |
| **65+** | black | Female | 0.00087209 | 0.00026856 |
| **65+** | black | Male | 0.00099674 | 0.00049979 |
| **65** | white | Female | 0.00064624 | 0.00022857 |
| **65** | White | Male | 0.00082106 | 0.00030998 |

[1] Surveillance Research Program, National Cancer Institute SEER*Stat software (seer.cancer.gov/seerstat) version 8.4.3.
